# Comparison of health-seeking characteristics of German and Belgian university students

**Published:** 2017

**Authors:** R Koop, H Kartounian, D Devroey

**Affiliations:** * Department of Family Medicine and Chronic Care, Vrije Universiteit Brussel, Belgium

**Keywords:** primary health care, Germany, Belgium, emergency care

## Abstract

**Introduction.** The aim of this study was to determine whether the use of primary health care differs between students enrolled in Belgian and German government-funded universities. The secondary aim of the study was to determine the factors that might explain such a difference.

**Methods.** Participants were recruited through all Belgian and German government-funded universities. Because not all the universities agreed to participate, recruiting was also done through social media groups of the universities. An anonymous online survey was used for data collection.

**Results.** In total, 2238 completed surveys were evaluated, of which 544 from students in Belgium and 1694 from students in Germany. In Belgium, more students had a family physician (87%) as compared to the students in Germany (73%) (p < 0.001). During the two months prior to the study, 37% of the Belgian students and 35% of the German students attended a family physician (p = 0.37). More German students attended a specialist (40%) as compared to the Belgian students (24%) (p<0.001). The German students also attended the emergency department more frequently (6%) as compared to their Belgian counterparts (3%) (p = 0.004).

**Conclusion.** Belgian university students were more likely to attend a primary care physician than the German students. The health care seemed to be better organized for Belgian students and they were more satisfied with the delivered care.

## Introduction

Students form a particular segment of the population when it comes to healthcare. They attend - often out of hours - for medical conditions related to their lifestyle. Thereupon, differences might exist in their health seeking behavior according to their health insurance, the country they study in and many other parameters. For this study, we were especially interested in Belgian and German students.

Health insurance

In Belgium, there is only one system of health insurance. The Belgian national health insurance (Rijksinstituut voor Ziekte- en Invaliditeitsverzekering) covers almost all Belgian inhabitants. However, Belgians can opt for an additional private insurance.

In Germany, there are two systems of health insurance: the “law-enforced” health insurance (Gesetzliche Krankenversicherung) and the “private” health insurance (Private Krankenversicherung). Approximately 70 of the 81.8 million inhabitants of Germany are insured by the “law-enforced” health insurance. The German authorities define who can apply for the law-enforced health insurance in the “Sozialgesetzbuch V”. The remaining 12 million Germans are obliged to apply for a private health insurance.

Choice of healthcare provider

In Belgium, the free choice of healthcare providers is defined in the 2002 law on the patients’ rights.

Patients are free to choose and change their FP, specialist, hospital, and emergency department. There is only an exception for military personnel, prisoners, life-threatening emergencies, people with mental disorders and patients in a care pathway.

In Germany, a law on the patients’ rights was approved in February 2013. However, the law is a collection of legal terms and the patients’ rights are very difficult to list. The free choice of the healthcare provider for example is not included in the law. On the other hand, the “Sozialgesetzbuch V” provides some information on the free choice of the healthcare provider. The insured are free to choose a healthcare provider as long as that healthcare provider accepts the legal and financial conditions imposed by the government. The insured are not supposed to change from the healthcare provider. However, they can change at every three months, but only for valid reasons. Patients can only choose a hospital when the referral letter does not mention a hospital. There is no free choice for the military. There are almost no restrictions for patients with private insurances.

*Health-seeking behavior*

Both countries organize health surveys to evaluate the health status of their inhabitants on a regular base, but also their health seeking behavior. In Belgium, this is done at every 3 to 4 years by the Scientific Institute of Public Health. In Germany, it is done every year by the National Association of Statutory Health Insurance Physicians [**1**,**2**]. Both surveys use a representative sample of the general population and do not focus on students. 

Students are in general younger and healthier than the general population; they attend for other medical conditions and have a different health-seeking behavior. For these reasons, we cannot use the results of the surveys to compare the health-seeking behavior of students. Neither do the results of the two surveys allow a comparison between the two countries. 

The aim of this study was to compare the use of the primary health care between the students enrolled in the Belgian and the German government-funded universities and to determine the factors that might explain the difference. 

## Methods

Questionnaire

The survey was done by an online questionnaire by using LimeSurvey. We chose LimeSurvey because there were no limits on the number of participants and we could make the questionnaire available in four languages (Dutch, French, German, and English).

The questionnaire was based on the 2008 questionnaire of the Belgian Health interview Survey (HIS) [**1**]. This survey was designed to provide data on the association between the socio-economic status and the health among different groups in the Belgian population. Only the questions related to the use and the accessibility of health care were selected from the existing survey.

The self-reported health status was indicated on a visual analogue scale (VAS) where the participants could score their perceived health status on a scale between 0 and 10 with 0 corresponding with the worst health participants could imagine, and 10 with the best health they could imagine. Such a VAS scale is used in the EQ-5D questionnaire [**3**]. However, in the EQ-5D, the scale ranged from 0 to 100 and not from 0 to 10.

The satisfaction with several healthcare providers was recorded as having a score from 1 to 5 with 1 corresponding to the worst healthcare service participants could imagine and 5 to the best healthcare service they could imagine.

Recruitment

The target population consisted of 1,546,136 students in Germany and 177,523 students in Belgium (88,794 in Dutch-speaking universities and 88,729 in French-speaking universities). 

A required sample size of 384 students was calculated for a population of 1,8 million students, a confidence interval of 95% and an error level of 5%.

The recruitment took place via the secretariats of the different universities. However, because of the privacy reasons and an abundance of surveys for which the cooperation of the students was asked, most of the universities refused to mail the survey to their students. Some of them published the link to the survey in the newsletter of their university. Nevertheless, for most universities, the recruitment was done through messages on social media. Some universities posted a message on their official social media pages. In other universities, the messages were posted on the social media pages of the student associations. Because we did not know how many universities posted the message, we did not have the exact figures on the number of students who were invited to participate in the survey.

The survey was made available online on February 12, 2013. The extraction of the data was done on April 22, since the required number of students was reached and the number of new responses was less than 1 per day for more than one week.

Inclusion- and exclusion-criteria

Only students enrolled in the Belgian or German government-funded universities could participate in the study. Nationality was not a selection criterion. Although Belgium as well as Germany has army-based universities that are funded by the government, students from these universities were excluded because they did not have a free choice for their medical care.

Statistics

Crude data, means, and standard deviations were available from LimeSurvey. Several control measures ensured the data quality. Data cleaning was performed in two steps: detection of errors in the dataset and correction of these errors. The data cleaning software looked for the missing data, systematic repetitive answers, discordant answers and errors related to the misinterpretation of questions. Errors were detected by using descriptive statistics, scatter plots, and histograms. The presence of systematic repetitive answers was considered when the same option was entered systematically in consecutive questions. 

For the mean distance, the patients travelled to receive medical aid, the outliers were not taken into account because they skewed the means considerably.

The statistical processing was done with OpenEpi 2.3.1, an open source software for epidemiologic statistics. Two by two tables were used to evaluate the relationship between two dichotomous variables by means of a Chi-square test. The Fisher exact test was used when at least one expected value (row total x column total/ grand total) was below five. The T-test was used to compare the means of two independent samples. 

## Results

Participants

In total, 2346 students participated in the study. One hundred and eight questionnaires were dropped because some questions were not completed. Two questionnaires were dropped because of deliberately misleading answers. Finally, 2238 valid questionnaires were retained: 544 (24%) from Belgian and 1694 (76%) from German universities. In Belgium, students from 6 of the 10 universities were recruited. In Germany, students from 60 of the 80 universities participated. Most of the participants were women: 68% in Belgium and 71% in Germany (p = 0.19). In Belgium, most respondents came from (para)medical students (41%), whereas in Germany most respondents came from educational sciences and psychology (21%).

Health insurance

In Belgium, 90% of the participants are insured through the Belgian health insurance, 8% through a non-Belgian insurance, and 2% by both a Belgian and a non-Belgian insurance. Three participants had no health insurance.

In Germany, 85% of the participants were insured through the law-enforced health insurance, 14% through a private insurance, and 1% through a non-German insurance. In Germany, none of the students was without insurance.

Health status

The German students evaluated their physical health status (78.9 SD 13.8), which was higher than the Belgian students (76.2 SD 12.6) (p < 0.001). The German students also evaluated their mental health status (85.1 SD 15.1), which was higher than the Belgian students (80.2 SD 14.1) (p < 0.001). 

Family physician

In Belgium, more students had a FP (87%) as compared to the students in Germany (73%) (p < 0.001). The most important reason for not having a family physician, met in both groups, was “I’m never ill” (Belgium 34%, Germany 52%). Among the Belgian students, the second most common reason (25%) for the absence of a FP was the fact that different FPs were consulted according to the problem. For the German students, other important reasons for not having a family physician, were that the students directly consulted a specialist (29%) or that they were absent for a long period (24%). Significantly, more German students complained that they could not find a good FP or that there was a lack of confidence (Germany 5% and Belgium 2%) (P = 0.025).

Within the 2 months before, 37% of the Belgian students and 35% of the German students visited a FP (p = 0.37). The mean number of contacts with a FP was higher among the German students (1.65 SD 1.08) than among the Belgian students (1.40 SD ± 0.88) (P < 0.001). Most of the students attended for a new problem (50% in Germany and 63% in Belgium) (P < 0.001).

Specialists

Within the 2 months before, more German students (40%) than Belgian students (24%) visited a specialist (p < 0.001). The mean number of contacts with a specialist was not higher among German (1.82 SD 1.92) than among Belgian students (1.74 SD 2.19) (P = 0.41). The most important reason to attend a specialist was a new problem for the Belgian students (44%), whereas the German students attended more for the follow-up of an existing problem (44%) (P < 0.001). A similar proportion of German students (29%) and Belgian students (25%) attended the specialist without a referral letter from a FP (p = 0.30).

Emergency care

Within the 2 months before, more German students (6%) than Belgian students (3%) visited an emergency department (p = 0.004). More German students (68%) than Belgian students (65%) attended the emergency department on their own initiative (p = 0.012). Only 9 German students and one Belgian student were referred to the emergency department by their FP (p = 0.29). 

The urgent character or the severity of the problem were the most important reasons for German (66%) and Belgian (77%) students to visit the emergency department instead of their FP (p = 0.048). More German students (62%) than Belgian students (35%) indicated that they attended the emergency department because the practice of the FP was closed (p = 0.001).

Medical service provisions on campus

Significantly, more German students (64%) were not informed about the medical facilities on their campus than the Belgian students (38%) (p < 0.001). Only 2% of the Belgian students as compared to 59% of the German students declared that there were no medical facilities on their campus (p < 0.001).

For their medical care, the Belgian students could attend a FP (77%), a hospital (59%), an emergency department (53%), or a first aid post (41%). The German students could attend a hospital (36%), an emergency department (30%), a FP (16%), or a first aid post (12%). The German students could attend significantly less medical service provision than the Belgian students (p < 0.001).

For the German students, the distance to the nearest FP was 11.4 km (SD 25.3). This was further as compared to 2.3 km (SD 3.3) for the Belgian students (p < 0.001). Also, the distance to a first aid post was less far in Belgium (1.9 km SD 2.2) than in Germany (2.4 km SD 2.2) (0.007). For the German students, the nearest hospital was closer (3.5 km SD 2.7) than for the Belgian students (4.2 km SD 4.0) (p < 0.001). Also, the nearest emergency department was closer for the German students (3.4 km SD 2.7) than for the Belgian students (4.0 km SD 3.8) (p = 0.014).

Satisfaction with healthcare

The Belgian students gave the emergency departments a higher score (3.5 SD 1.0) than the German students (3.1 SD 1.1) (p < 0.001). The Belgian students were also more satisfied with the service of FP (4.1 SD 0.8) than the German students (3.9 SD 0.9) (p = 0.008).

Specialists working outside hospitals received a similar score from the Belgian students (3.8 SD 0.8) than from the German students (3.7 SD 0.9) (p = 0.54). However, the Belgian students were also more satisfied with the service of specialists working inside hospitals (3.7 SD 0.9) than the German students (3.3 SD 1.0) (p < 0.001).

## Discussion

Health insurance

In Belgium (and not in Germany), some students do not have a health insurance. These students are foreign students both from inside the European Union and from outside the Union. Students who wish to subscribe to a Belgian university do not have to prove that they have a health insurance. However, the enrolment in an institution of higher education does not give students the right to join the Belgian health insurance system.

The absence of students without a health insurance in the German group could be explained by the fact that at enrolment in a German university, students need to prove that they have a health insurance. This may be a law-enforced or a private insurance. This regulation also applies to foreign students.

Health status

German students estimated their physical and mental health status higher than Belgian students did. These values were slightly below the OECD values for the general health status of the year 2011 with 86% for Belgium and 81% for Germany in the age-group of 15 to 24 years [**4**]. However, we could not statistically compare our figures with those of the OECD because the OECD did not provide standard deviations and did not make a distinction between the physical and the mental health. However, the estimation of health was higher in Belgium than in Germany, while the students in Germany estimated their health higher than in Belgium. 

Regular family physician

The German students were significantly less likely to have a regular FP than Belgian students. The German students also score lower as compared to the general German population (94%) and the population under 35 years with a high level of education (88%) [**5**]. According to the Belgian HIS, 95% of the Belgian population has a regular FP [**1**]. Both in Germany and in Belgium, students are less likely to have a regular FP as their peers of the same age.

The most important reason for not having a FP was the fact that the students declared to have never been ill. The higher proportion of the German students for not having a regular FP was in line with their higher self-reported health. Another reason could be the fact that more German parents attended a paediatrician for their children than the Belgian parents. Paediatricians in Germany, and not in Belgium, belong to the primary healthcare. The greater distance to their FP might also play an important role for German students. From our study, it was not clear whether students remained loyal to their local FP in their hometown or took a new FP during their studies in another city. 

Patient encounters

German students consulted their FP more frequently for a known problem, whereas the Belgian students consulted the FP for new problems. The follow-up of known problems could explain the higher mean number of consultations with their FP among the German students. However, it was also possible that the German FPs recalled their patients more than the Belgian FPs did. On the other hand, it could be related to the shorter time the German FP spent on a consultation. A German FP spends normally 8 minutes per patient encounter [**6**]. A Belgian FP spends normally 15 minutes per patient encounter [**7**]. It is well known from the OECD figures that German patients attend a physician more frequently (8.9 times/ year) than Belgian patients do (7.7 times/ year) [**4**]. It is plausible to believe that the German FPs cannot handle the problem(s) within 8 minutes and for that reason, they back order their patients more frequently. The fact that Belgian students have to pay their FP after every consultation and the German students do not have to pay their FP, may also play a role in the number of contacts. Maybe all these factors declare why German students have it more difficult to find a suitable FP. 

**Table 1:** Reason for encounters with a family physician

	Belgium (n=199)	Germany (n=584)
One or more NEW diseases, complaints or health problems	63%	49%
Control or follow-up for KNOWN diseases, complaints, or health problems	26%	37%
You didn’t have a disease, complaint or health problem, but consulted the GP or the family doctor for another reason	10%	13%
I do not know	1%	1%

Specialists

Despite that, the German students estimated their health status higher than the Belgian students, consulting a specialist significantly more. It is less likely that their health status was higher “because” of the higher consumption of specialist care. Another reason for the higher number of contacts with a specialist in Germany was the higher referral rate from FP to specialists. Another important reason for the high proportion of specialist care could be the fact that the German patients with a law-enforced insurance pay the same fee for specialists as for FP.

**Table 2:** Reason for encounters with a specialist

	Belgium (n=129)	Germany (n=672)
One or more NEW diseases, complaints or health problems	30%	49%
Control or follow-up for KNOWN diseases, complaints, or health problems	44%	37%
You didn’t have a disease, complaint or health problem, but consulted the GP or the family doctor for another reason	25%	13%
I do not know	1%	1%

Emergency care

German students visited the emergency department almost twice as much as compared to the Belgian students. The most credible reasons for the high amount of visits to the emergency department in Germany were the short opening hours of the GP and the fact that the emergency departments were located closer to the university than the FP. What needs to be mentioned is that out-of-hours services are provided by FPs in both countries. 

Medical service provisions

More German than Belgian students were not informed regarding the medical care provisions on their campus. It was not clear whether the students were not informed about the provisions or if there were no provisions at all. However, it seemed unlikely that two thirds of the German universities had no medical provisions.

From the distances to the medical provisions, we understood that a dense network existed in both countries. The long distances and high standard deviations to FPs in Germany made us believe that many students indicated their home FP, as their regular FP. Belgian students seemed to visit a new FP in the neighborhood of their university faster. This attitude might have also declared the higher number of visits to specialists and emergency departments. 

Satisfaction with healthcare

The Belgian students were more satisfied with the care provided by the emergency departments, FPs, and specialists working in the hospitals, than the German students. It was also remarkable that both German students and Belgian students gave the highest score to their FP and the lowest score to the emergency department.

Limitations of the study

An online survey is a method with several limitations. The questions should be clear and everyone should understand the questions in the same manner. Moreover, the respondents can invent fictitious answers and data. Our online questionnaire was not tested in a pilot study. A pilot study might have detected some shortcomings in the questionnaire. At the beginning of the registration, some shortcomings were reported by participants. The questionnaire was adapted where possible and without changing the context.

## Conclusion

Students in Germany estimated their physical and mental health better as compared to Belgian students. More students in Belgium have a regular FP, while German students have more contacts with other physicians, especially specialists.

Both in Belgium and Germany, fewer students have a regular FP than the general population of the same age group. German students mainly visit a physician for long-known problems, while Belgian students attend them for new problems. 

German students attend the emergency departments more often, mainly because their FP is not available for consultations. 

Belgian students are better informed about healthcare provision in their campus, and health care seems to be better organized in Belgian universities. Distances to FP are longer in Germany but hospitals and emergency departments are closer to the university. Satisfaction about health care services is higher in Belgium than in Germany.

**Acknowledgement**

The authors would like to thank all students who participated in the survey and David Proot for the English language editing.

**Declaration of interests**

The authors have no financial or personal relationships with other people or organizations that could inappropriately influence their work. There are no conflicts of interest.

**Role of the funding source**

The study was not funded.
